# Childhood trauma, depression, and the risk of incident prediabetes in young adults: findings from the Lifelines Cohort Study

**DOI:** 10.1093/eurpub/ckac129.700

**Published:** 2022-10-25

**Authors:** S Deschenes, F Nearchou, A McInerney, N Schmitz, A Nouwen

**Affiliations:** 1 University College Dublin, Psychology, Dublin, Ireland; 2 Tuebingen University, Population-Based Medicine, Tuebingen, Germany; 3 McGill University, Psychiatry & Epidemiology, Montreal, Canada; 4 Midlesex University, Psychology, London, UK

## Abstract

**Background:**

Childhood trauma and depression have been shown to increase the risk of type 2 diabetes. However, many studies have focused on middle-age and older adults, with less known on the role of these variables in early glucose dysregulation. The goal of the study was to examine childhood trauma, depression, and their interactions, as risk factors for the onset of prediabetes in young adults.

**Methods:**

Data were from the Dutch Lifelines Cohort Study. N = 8,650 adults (61% female) between 18-35 years without prediabetes/diabetes at baseline (2007-2014) were included. Childhood trauma was assessed using the Childhood Trauma Questionnaire. Depression was assessed using the Mini International Neuropsychiatric Interview. Prediabetes at follow-up (2014-2017) was considered by haemoglobin A1c levels between 5.7%-6.4%. Logistic regressions examined associations between depression and childhood trauma with the risk of incident prediabetes. Odds ratios (OR) and 95% confidence intervals (CI) for unadjusted analyses and analyses adjusted for age, sex, education, ethnicity, body mass index, smoking, and alcohol use (reduced adjusted sample size; n = 7,186) are presented.

**Results:**

244 participants (2.8%) developed prediabetes. In univariate analyses, childhood trauma (OR = 1.02, CI = 1.01-1.03, p=.006) and depression (OR = 2.08, CI = 1.01-4.29, p=.048) predicted incident prediabetes. When childhood trauma subscales were examined, only sexual abuse significantly predicted incident prediabetes. In adjusted analyses, only childhood trauma, specifically sexual abuse, significantly predicted incident prediabetes (OR = 1.06, CI = 1.01-1.12, p=.021). No multiplicative interaction between depression and childhood trauma was found.

**Conclusions:**

Young adults who have experienced childhood trauma, particularly sexual abuse, may be at risk of glucose dysregulation in early adulthood. Early targeted preventive care may help attenuate or halt glucose dysregulation and the development of type 2 diabetes.

